# Cancer becomes wasteful: emerging roles of exosomes^†^ in cell-fate determination

**DOI:** 10.3402/jev.v2i0.22390

**Published:** 2013-09-24

**Authors:** Franz Wendler, Neus Bota-Rabassedas, Xavier Franch-Marro

**Affiliations:** 1 past address: CNRS UMR, Inserm UMR, Institute de Biologie Valrose (IBV), Centre de Biochemie, Nice, France; 2 CSIC, Institute of Evolutionary Biology IBE CSIC-UPF, Barcelona, Spain

**Keywords:** multivesicular body (MVB), extracellular vesicles, exosomes, cell-fate ligands, secretion, signalling pathways

## Abstract

Extracellular vesicles (EVs), including exosomes, have been widely recognized for their role in intercellular communication of the immune response system. In the past few years, significance has been given to exosomes in the induction and modulation of cell-fate-inducing signalling pathways, such as the Hedgehog (Hh), Wnts, Notch, transforming growth factor (TGF-β), epidermal growth factor (EGF) and fibroblast growth factor (FGF) pathways, placing them in the wider context of development and also of cancer. These protein families induce signalling cascades responsible for tissue specification, homeostasis and maintenance. Exosomes contribute to cell-fate signal secretion, and vice versa exosome secretion can be induced by these proteins. Interestingly, exosomes can also transfer their mRNA to host cells or modulate the signalling pathways directly by the removal of downstream effector molecules from the cell. Surprisingly, much of what we know about the function of exosomes in cell determination is gathered from pathological transformed cancer cells and wound healing while data about their biogenesis and biology in normal developing and adult tissue lag behind. In this report, we will summarize some of the published literature and point to current advances and questions in this fast-developing topic. In a brief foray, we will also update and shortly discuss their potential in diagnosis and targeted cancer treatment.

## Gloss

Most of us have to declutter sometimes, pack things into bags before giving them away, but sometimes we do not get rid of those things. Cells also dispose of unwanted proteins in bags (vesicles) and also under some conditions reuse them to communicate with their environment. Small vesicles called exosomes can carry out both of these functions. They are generated within specific compartments of the cell called multivesicular bodies (MVBs) or endosome-like plasma membrane microdomains and then released from the cell. Exosomes carry a broad number of cargo, including cell-fate-promoting signalling molecules. Such molecules take part in determining cell identity, and deregulation of the induced signalling pathways is related to many forms of cancers. Interestingly, it has been shown that exosome-bound cell signals can shape the microenvironment of cancer cells or induce wound-healing processes. For that reason and due to their cell-type-specific composition, stability and easy accessibility from body fluids, exosomes have great potential to become valuable determinants in the diagnosis and targeted treatment of diseases such as cancer. This review will summarize and discuss some of the recently published data.

## Introduction

Multivesicular bodies (MVBs) form an endosomal-intermediate compartment directing endocytosed cargo for lysosomal degradation. Surprisingly, pulse-chase and electron microscopy studies of transferrin receptor trafficking in reticulocytes in the 1980s, demonstrated that MVBs are also able to fuse back to the plasma membrane, releasing small vesicles that were later named “exosomes” (1, 2). Exosomes are defined as lipid-bilayer-enclosed vesicles with a diameter of approximately 50–100 nm that are either released from secretory MVBs upon fusion with the plasma membrane or pinched off from endosome-like plasma membrane domains [virtual spaces: www.isev.org; www.facebook.com/groups/exovesicles/; (3)]. While exosomes are formed from many proteins that are common to all types of exosomes, they are also composed of proteins that show cell-type specificity (http://evpedia.info; http://www.microvesicles.org). Research into extracellular vesicles (EVs) underwent 2 major breakthroughs in the last 3 decades. Originally, exosomes were considered as garbage bags used to discard obsolete cytosolic and membranous molecular remnants, however in the mid-1990s with the discovery of antigen-presenting EVs from B-lymphocytes, they have been associated with immune system functions (4). Since then, the focus of exosome research has concentrated on the physiological role of exosomes in the immune response, particularly in the interaction of immune and cancer cells [extensively reviewed in (5–11)]. The second big leap, in the first decade of this century, brought the discovery of miRNA and mRNA as exosomal cargo (12). In recent years, a plethora of reports have revealed another function of exosomes: they work as intercellular communication shuttles transporting signalling molecules, including cell-fate proteins (13).

**Fig. 1:**
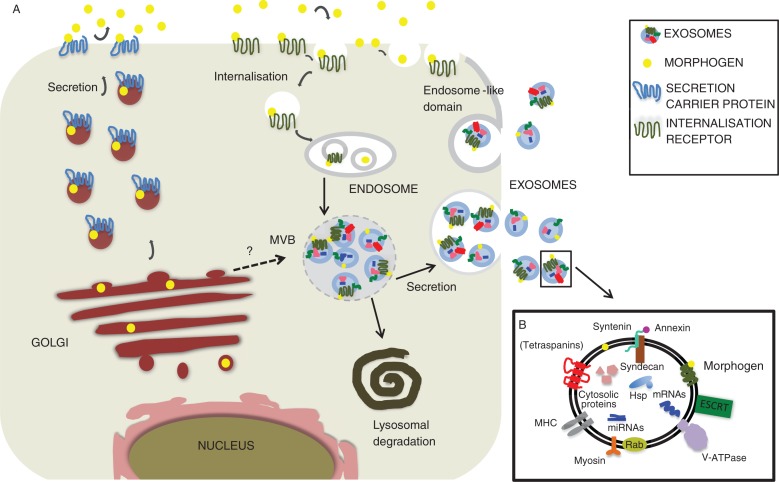
Hypothetical representation depicting putative cell-fate-determinant secretion pathways that could lead to exosome secretion. (A) After synthesis and putative modification a generic signalling ligand transits to the plasma membrane and from there becomes re-internalized either alone or through receptor-mediated endocytosis. After passage through the endosomal network, it eventually undergoes inclusion into MVBs. MVBs can then either fuse with the lysosome leading to signal degradation or become secretory MVBs that fuse with the plasma membrane and release exosome-bound signalling ligands. Such proteins, however could also be directly transported to the endosomal compartment after synthesis and included into endosomes. Alternatively, exosomes could be generated at the plasma membrane itself in endosome-like domains. Signalling ligands are represented as yellow circles. (B) Summarizing components found to carry out functions during exosome trafficking.

Many cell-fate-determining ligands are evolutionary conserved, mostly secreted proteins that induce target gene expression according to their local concentration by binding to their respective receptors localized on responding cells [reviewed recently in (14)]. Once released from the place of production, they are transported through the extracellular environment of adjacent tissues, forming spatial and temporal gradients, orchestrating morphogenesis, growth, differentiation and adult tissue homeostasis. Hedgehog (Hh), Wnts, Notch, TGF-β, EGF and FGF constitute the major cell-fate proteins. Wnt and Hh ligands are lipid-modified and traffic to the plasma membrane together with their own dedicated receptor Evi also known as sprinter, Wntless or Gpr177 (15, 16) and dispatched (17), respectively, whereas TGF-β ligands have N-terminal secretion signal sequences (18, 19). In addition, EGF ligands have to be proteolytic processed before they are released, whereas the Notch ligand Delta is a transmembrane protein that needs cell–cell contact to activate its signalling pathway. However, all of them have been found in exosomes, although it is currently unclear how these proteins become exosomal cargo. There are at least 2 possibilities, either they could be directly sorted into MVBs after synthesis or become packaged into secretory MVBs after re-internalization (Fig. 1A).

Deregulation of any of the secretory pathways may alter signalling activities, leading to severe pathogenesis, including neurological diseases and cancer. This review will focus on the effects of secreted cell-fate proteins using the exosomal pathway. However, caution should be taken as the term “exosome” in some reports might refer to an operational use (e.g. vesicles obtained by high-speed ultracentrifugation) rather than to the cell-biological origin (3). In addition, exosomes might not always originate from MVBs, as for instance T-cell plasma membrane can release HIV Gag protein from exosomes that bud from endosome-like plasma membrane domains [(20); also see below]. Finally, it has been proposed that type III unconventional protein secretion bypassing the Golgi apparatus may generate one kind of secretory MVBs that release exosomes or exosome-like particles into the extracellular space (21, 22). Interestingly, the latter mechanism of EV production shares in its early state proteins involved in autophagy, is linked to nutrient supply, and therefore to external cues. All in all, in this report we speak of “exosomes” about vesicles, which according to the described studies derive from endosome or endosome-like organelles or domains.

## Biogenesis, cargo sorting, secretion and reception of exosomes

Once generated, MVBs can either fuse with the lysosome or the plasma membrane, thus releasing vesicles. What distinguishes secretory from degradative MVBs is still a conundrum. Proteomic cataloguing however, show some overlapping similarities in the profiles of secretory and degradative exosomes including membrane and cytosolic proteins (http://www.microvesicles.org/; Fig. 1B). There is a plethora of data available for the role of endosomal sorting complex required for transport (ESCRT) proteins in the generation of both degradative and secretory MVBs [reviewed in (22–24)]. ESCRT proteins help in cargo selection and the inward budding process (away from the cytoplasm). Some of the components of the ESCRT complex such as VPS31, VPS4B and TSG101 have been localized to endosome-like plasma membrane domains that generate exosomes (20). In addition, the ESCRT-0 component Hrs affects the intracellular production of MVB-derived exosomes in dendritic cells and syndecan, syntenin and Alix proteins have been found to profoundly impact on exosomal FGF trafficking (25–27). However, Trajkovic and colleagues did not find any involvement of ESCRT proteins in the formation of exosomes. In contrast, they did find that ceramide (a sphingolipid) triggers inward budding to produce exosomes (28). These discordant data might reflect a cell-type specificity and diversity of mechanisms that control the generation of exosomes. Additional research is needed to define the core machinery that constitutes secretory MVBs.

Once secretory MVBs are formed they have to fuse with the plasma membrane. Different proteins are involved in this process, but only a few have been recently related to cell-fate ligand-bound exosomes. Rab GTPases that regulate many steps in membrane trafficking are among them. For instance, Rab11 (known as a recycling endosome Rab protein) has been shown to enhance Wnt-exosome secretion in *Drosophila* S2 cells and is involved in the release of Wg/Evi complex from synaptic boutons of the neuro-muscular junctions (NMJs) in developing *Drosophila* larvae (29, 30). These data are in accordance with earlier results obtained from K562 cells that need Rab11 and calcium for MVB fusion with the plasma membrane (31); oligodendrocytes use Rab35 and its GTPase-activating protein TBC1D10A-C for docking and tethering of MVBs (32); and a siRNA screen in HeLa cells identified 5 Rabs, Rab27a and b among them, to promote exosome release (33). Fusion of secretory MVBs that are generated along type III unconventional protein secretion requires the SNARE protein Sso1 (34). Another fusion machinery component might be the V0 section of the vacuolar H ^+^-ATPase (V-ATPase) as evidence suggested that it possibly participates in exosomal secretion of Hh-related proteins (35). The same V-ATPase also takes part in synaptic vesicle exocytosis making it a candidate for Wnt-bound exosomes in the NMJs in *Drosophila* (36). Finally, Koles and colleagues revealed that release of Wnt exosomes at the NMJs in the developing *Drosophila* depended additionally to Rab11 also on the SNARE protein syntaxin 1A (also involved in synaptic vesicle exocytosis) and myosin 5 (30). All these data suggest a specificity of secretory MVBs, most likely depending on the cargo, and diversity of mechanisms involved in its formation.

Different proteins are transported through exosomes but sorting signals for cargo are poorly understood. It has been shown that tetraspanin family members might play a role in cargo sorting. Tetraspanin are membrane proteins that cluster with themselves and with other membrane and cytosolic proteins into tetraspanin-enriched microdomains (TEMs). Such TEMs may function in signalling and also intracellular trafficking processes (37). For instance, the 2 tetraspanins CD9 and CD82 both promote β-catenin (a Wnt signalling transducer protein) secretion via exosomes (38). Another tetraspanin, CD63, is routinely found in exosome profiles but its function remains widely elusive, although van Niel et al. show a role of CD63 in intraluminal sorting of a particular protein inside melanosomes (a type of lysosome-related organelle or MVB) (39). Additional sorting information might be given by anionic phospholipids. Several lines of evidence suggest that in T cells, membrane anchoring together with oligomerization, could in some cases be sufficient to direct proteins to exosomes budded from endosome-like plasma membrane domains or unconventional protein secretion (40, 41). In addition, Vidal et al. have shown a role for protein aggregation in directing proteins to MVBs (42). Wnt and Hh family proteins potentially classify for such a sorting mechanism, as their members are plasma membrane residents, dually lipid-modified (WntD being an exception) and functionally oligomerize. Nevertheless, experimental validation is not yet available. Wnts can also be secondarily secreted on exosomes; for instance, after internalization, synthetically generated Wnt3a, but not Wnt5a or Wnt5b, protein in microglia cultures becomes exosomally secreted (43). Finally, an additional layer of complexity is added as different exosome species might be released by one cell. For instance, human organoids derived from the colon carcinoma cell line LIM1863 can secrete 2 populations of apical and basolaterally produced exosomes containing different contents (44). Cell-fate proteins secreted from polarized epithelial cells differ in their secretion in that they can be secreted apically and basolaterally acting in short- and long-range signalling. Therefore, it will be interesting to determine whether exosome-bound signalling proteins contribute to any of these activities.

Upon loading and release, exosomes travel through the extracellular space and are eventually internalized by cells. How target cells are chosen is not well understood. Macropinocytosis, clathrin-dependent endocytosis and phagocytic mechanisms have all been discussed (45, 46). Different mechanisms might apply. Evidence exists also that at one point after uptake, exosomes accumulate with endosomal markers (47). In addition, the previously mentioned exosome-resident tetraspanins might contribute to target cell selection as well as glycoproteins (47, 48).

Most cell-fate proteins (bicoid transcription factor being an exception) unfold their signalling capacity through binding to specific cell-surface receptors. Therefore, it will be of great interest to identify a possible role the respective receptors might play in exosome–cell interactions.

## Bioactive role of cell-fate signals presenting exosomes in development and tissue repear

EV-bound Wingless (Wg, a *Drosophila* Wnt protein) can spread through wing imaginal disc epithelial tissue (49). Greco et al. called those vesicles “argosomes” (49). Later, the view of the nature of argosomes changed and they are now considered as monolayer, enclosed lipid particles (50). Nevertheless, these findings inspired many scientists in the field to revisit the ideas of how cell-fate-signalling proteins are secreted. A decision as to whether Wg (a Drosophila Wnt protein) bound to exosomes plays a role during *Drosophila* development is still pending. Gross et al. found that fractions of secreted Wg co-stained with the overexpressed exosomal markers CD63 (a tetraspanin) as well as with overexpressed Rab4 GTPase outside the Wg expressing domain in the wing imaginal disc of *Drosophila*, however the functional data suggested are not available (51). Beckett et al. concluded indirectly from knockdown experiments of the exosome secretion promoting Rab11 protein (see above), and from the absence of the Wg accompanying Evi receptor, that Wg exosomes most likely do not participate in wing disc gradient formation (29). Nonetheless, both groups found that *Drosophila* and human cell lines can release functional Wg(Wnt)-exosomes (29, 51). It will be a major challenge to answer the question of whether cell-fate proteins bound to exosomes indeed take part in tissue patterning during developmental processes.

Exosomes also play a part in tissue homeostasis such as wound healing. For instance, after injury, epithelial cells can increase the number of exosomes transmitting TGF-β1 mRNA, activating fibroblast differentiation during the repair and regeneration after parenchymal injuries (52). In other processes, exosome-mediated delivery of Sonic Hedgehog (Shh) to ischemic myocardium was also shown to enhance stem cell activities towards preservation of normal cardiac function (53). In addition, evidence also showed that signalling factors, such as EGF, can induce the production of ErbB1 receptor-containing exosomes in the keratinocytes cell line HaCaT (54). Despite the importance of these results, to date physiological data remain sparse. Most studies were conducted using unphysiological exosome concentrations, whereas in vivo investigations of exosomal-induced mechanisms are hampered by the lack of insight into their biogenesis.

## Role of signalling exosomes during cancer formation

**Fig. 2:**
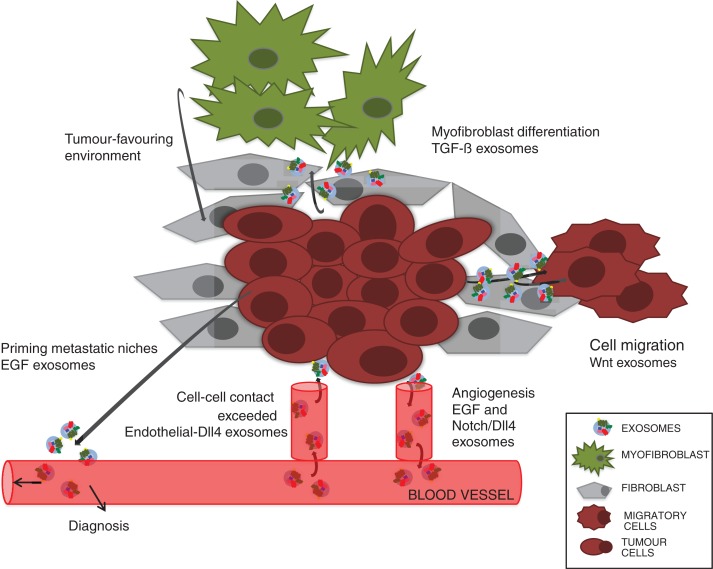
Schematic representation of a hypothetical microenvironment summarizing cell communication through cell-fate signals carrying exosomes.

It is well established that exosomes can induce an adaptive immune response in the vicinity of cancerous cells by promoting or suppressing immunoactivities (7). However, ample data also suggest that cell-fate signals bound to exosomes can induce profound changes within the microenvironment of a developing tumour (Fig. 2). As discussed earlier, signalling ligands provide one kind of input important for tissue homeostasis. To better understand the data discussed below, here is a short introduction into cancer biology.

One of the hallmarks of cancer cells is that they react with their microenvironment, which provides the interface between parenchymal tumour cells and surrounding stroma. The latter consists of normal non-transformed sibling cells, blood-vessel-forming cells, immune and inflammatory cells and mesenchymal stem cell (MSC)-derived fibroblasts. For tumour suppression or progression, cross-talk between the different components of the microenvironment is needed. Such communication is executed by growth factors, including chemokines and cytokines and cell-fate-signalling molecules. Although the intrinsic variability of the microenvironment of tumours might produce different outputs, it seems clear that exosomes play an important role in this exchange of information.

## Cell-fate ligand-bound exosomes help to shape the cancer cell microenvironment

As mentioned earlier, vascularization provides a means of transport of nutrients to tumour growth sites (Fig. 2); in progressive stages of cancer development tumours can become invasive and metastasize. Recent experiments have shown that surface-presenting Shh microparticles and exosomes take part in the modulation of this multistep process (55). Human breast and colorectal cancer cells release exosomes carrying EGFR-ligands (EGF, TGF-α, amphiregulin) that might promote priming of metastatic niches (56). In addition, in a mixed human and mouse experimental system, Wnt11 exosomes activate the Wnt-planar cell-polarity signalling pathway in breast cancer cells (BCCs), allowing the formation of protrusions necessary for cell migration (57). Interestingly, while Wnt11 derives from the BCCs themselves, the exosomes derive from stroma-resident fibroblasts. In some cases, exosomes can also induce cell changes in the stroma. For instance, TGF-β exosomes released by mesothelioma cells can induce the expression of α-smooth muscle actin, a marker for fibroblast to myofibroblast transition. Myofibroblasts, in turn, provide a tumour-favouring environment important for tumour growth, vascularization and metastasis, but also play a role in wound healing (58). Cancer-derived TGF-β exosomes induce tumour evasion through their anti-proliferative effects on blood lymphocytes in the vicinity of cancer cells (59). Conversely, exosomes from breast cancer cells can have an impact on myofibroblast differentiation from mesenchymal stem cells, accompanied by the increased expression of tumour-promoting factors, including VEGF and TGF-β (60). Another mechanism of how exosomes from cancerous cells can cause phenotypic changes in their proximity is exemplified by Notch. Both Notch and its ligand Delta-like 4 (Dll4) play an important role during neo-vascularization and angiogenesis. Dll-4 becomes upregulated in endothelial cells resulting in its incorporation into endothelial-derived exosomes, leading to inhibition of Notch signalling beyond cell–cell contact (61).

Finally, autocrine-produced EVs might determine tumour cell-fate itself. For example, exosomes produced by human pancreatic tumour cell lines can hamper their own Notch survival pathway leading to apoptosis (62).

It has recently been shown that exosomes can also modulate the strength of signalling through the cellular export of downstream effector molecules. In this way, cells can attenuate canonical Wnt activity through the removal of the downstream effector β-catenin (38). In this context, it is interesting to note that during Wnt signalling β-catenin is required for GSK3 sequestration to MVBs (63). How these two findings potentially synergize in Wnt signalling regulation still needs to be addressed. Exosomal secretion of the Wnt-antagonist Dickkopf-related protein 4 (DKK-4) might also regulate Wnt signalling (64). This was concluded indirectly as epithelial colon cancer cells lose exosomal DKK-4 regulation of Wnt signalling during colorectal cancer progression (64).

In summary, the examples described above show just how important and versatile exosomes are in all stages of cancer generation. Associated with cell-fate-determining proteins, they can cause profound cell changes and therefore shape the pathological transformed microenvironment. As exosome composition seems to be cell and tissue specific, they are highly suitable to serve as diagnostic markers.

## Exosomes as biomarkers in diagnosis

During cancer progression to more aggressive phenotypes, cancer cells alter their molecular signature causing quali- and quantitative changes of exosomes that in turn affect their (micro-) environment. These properties, together with exosomes’ stability and easy accessibility from body fluids such as blood, urine, cerebrospinal liquid, saliva or breast milk amongst others, label them as ideal biomarkers. For instance, as tumours become more aggressive, heparanase activity is enhanced in myeloma, lymphomas or breast cancer, increasing both the number of exosomes released and the amount of syndecan-1, VEGF and HGF molecules exposed on their surface (65), making them more available for detection. Heparanase modifies cell surface (and expectantly vesicle surface) and extracellular matrix, such as heparan sulfate proteoglycans (HSPGs), having an impact on angiogenesis, invasiveness and metastasis (66). Therefore, exosome profiling is highly likely to aid the determination of the malignancy and stage of a particular cancer (67, 68). Thus, the high-throughput profiling of signalling exosomes from different forms and stages of cancer potentially holds huge information promise for pre-stage diagnosis and cancer stage-specific intervention.

## Potential of exosomes in targeted cancer treatment

Exosomes could be considered as naturally occurring liposomes (artificially generated bilayer enclosed vesicles) carrying proteins, such as antigens, mRNA and microRNA. Liposomes have long been thought of as a delivery system for therapeutics (69). One feature of exosomes similar to liposomes could be their ability to cross the blood–brain barrier, making them a true systemic signalling carrier (70). Research has to be conducted to validate this point. For instance, the exosome signature obtained from patients with glioblastoma revealed a high content of TGF-β and tumour antigen EGFRvIII, amongst others (70–72).

How can exosomes be considered as cancer therapeutics? Since exosome identity depends on the cell type, one way would be to load the inner vesicular space of signalling exosomes with biological or chemical agents. That could be done, for instance, by semi-synthetic exosomes produced by genetically designed cells and subsequent re-injection into the organism. A similar approach could be used to re-supply tissues with signalling molecules to overcome signalling deficits (regeneration after stroke or heart attack) or to help cell differentiation in vivo. In addition, the effects of chemical compounds on cancer exosome cell mixtures could be directly studied in high-throughput formats. These approaches will need a good understanding of the fundamental cell biology of exosome biogenesis.

## Conclusion

Thus, do we talk about garbage bags after all when we talk about exosomes? Much of the evidence presented here concerning their role in signalling seems to support this idea in a very particular way. For instance, cancer cells produce an abundance of upregulated miRNA, mRNA and proteins, with signalling ligands amongst them. Consequently, cells might use exosomal export as one route to get rid of this excess material. Cancer cells present a special physiological state, constantly modulating and adjusting their composition, competing with their neighbouring cells for a niche to settle in. All too convenient in this struggle might be the export of signalling ligands, which help to set and shape the microenvironment. We hypothesize that exosome-bound signals might be more stable in the extracellular space than free-accessible ones, bringing along additional factors and increasing local concentrations, all in all prerequisites to increase their chance to impact on distant cells. These properties and their easy access in body fluids are working in our favour as the quali- and quantitative composition of exosomes might report on the kind, progression and aggressiveness of a particular cancer, as has been reported in selected cases. Concluding from the presented data, exosomes could be utilized in at least 2 ways in cancer therapy: re-applying naturally or synthetically altered exosomes to the organism to target signalling, or by interception of any of the steps during the “life” of an exosome through specific drug intervention. Both ways will need intensive research into the fundamental cell biology of exosome biogenesis. Highly interesting for the biomedical community will be the question whether signalling exosomes hold systemic information for cancer cells to metastasize. Not least, investigating the functional role of exosomes as a carrier of signalling ligands in the developing and adult organism will remain as another major challenge for the future. The enticement to follow these threads lies just in front of us.
